# Impact of clinical history on choice of abdominal/pelvic CT protocol in the Emergency Department

**DOI:** 10.1371/journal.pone.0201694

**Published:** 2018-08-07

**Authors:** Wilfred Dang, Pawel D. Stefanski, Ania Z. Kielar, Mohamed El-Khodary, Christian van der Pol, Rebecca Thornhill, Arash Jaberi, Angel Y. N. Fu, Matthew D. McInnes

**Affiliations:** 1 Faculty of Medicine, University of Ottawa, Ottawa, Ontario, Canada; 2 Department of Radiology, The Ottawa Hospital, Ottawa, Ontario, Canada; 3 Ottawa Hospital Research Institute, Ottawa, Canada; Cedars-Sinai Medical Center, UNITED STATES

## Abstract

**Introduction:**

Radiologists and other specialty consultants play a role in diagnosing patients with acute abdominal conditions. Numerous Computed Tomography (CT) protocols are available and radiologists’ choices are influenced by the clinical history provided. We hypothesize that the quality of the initial communication between referring physicians and radiologists greatly affects the utilization of health resources and subsequent patient care. The purpose of this pilot study was to employ a grading system to quantitatively evaluate a provided history. We also sought to evaluate inter-rater reliability by having radiologists evaluate sample histories and finally, to assess whether the quality of history has an impact on the number of CT protocols radiologists choose as potentially appropriate, with less potential protocols being seen as a positive outcome.

**Methods:**

Four reviewers, (2 attendings and 2 residents) evaluated 350 consecutive clinical histories provided for patients presenting to a tertiary care Emergency Department (ED) between September–October, 2012. Reviewers graded histories on a 5-point scale using 4 categories of criteria. This includes a) presenting complaint, b) relevant past medical history or symptom evolution, c) objective laboratory or prior examination results and d) differential diagnosis.

**Results:**

There was substantial agreement among all four reviewers when evaluating the quality of history, ICC 0.61, (95% CI 0.48–0.71). In particular, agreement amongst attending radiologists was substantial, with ICC 0.69 (0.48–0.80). Significant negative correlation was observed between history grade and number of potentially appropriate protocols in 3 of 4 reviewers (Spearman’s rho: -0.394, -0.639, -0.864, p <0.0001 for these reviewers). This correlation was significantly stronger for attending radiologists (Spearman’s rho: -0.763, 95% CI -0.7933 to -0.731; p<0.0001). Agreement was poor among reviewers when asked exactly how many protocols could potentially be used to answer the clinical question based on provided history, ICC 0.08, (95% -0.03–0.13).

**Conclusion:**

Although there is still variability in radiologists’ approach to protocoling urgent studies, a more comprehensive requisition history narrowed the number of protocols considered.

## Introduction

Computed Tomography (CT) has become a commonly used imaging modality in the Emergency Department (ED) due its availability and diagnostic utility. In this institution’s country, there is also an increasing demand of imaging requests due to high ED patient volumes. This is compounded by the challenge of a need to reduce patient wait-times and healthcare costs, while still maintaining diagnostic efficacy and patient safety [1–3].

Communication in radiology has become a focus of many publications in recent literature. Much of this research has focused on accuracy and efficiency of communication from the radiologist reporting a final imaging interpretation [4–7]. However, the importance of the initial interaction and quality of history provided to the radiologists prior to imaging, particularly with abdominal imaging, has only been discussed in a few publications, with mixed results [8–12].

The quality of clinical history provided by the ED physician on an imaging requisition gives the radiologist context on the patient: The radiologist uses this to determine the appropriate imaging modality to use and the best protocol(s) to answer the diagnostic question. Each protocol utilized has differences in imaging acquisition technique. This includes patient positioning, presence or absence of intravenous or enteric contrast agents as well as differences in timing of scanning (and number of post-contrast scanning phases) after administration of intravenous or enteric contrast. The protocol chosen can affect a radiologist’s ability to make an accurate diagnoses. For example use of oral contrast can make diagnosis of ischemic bowel very difficult since the dense oral contrast in the bowel lumen reduces the ability to detect contrast enhancement of the adjacent bowel wall. Also, presence of intravenous contrast in the kidneys can mask small renal calculi, whereas lack of intravenous contrast will make diagnosis of a renal or splenic infarct impossible. In addition, the type of CT imaging protocol chosen for a clinical presentation can affect the amount of radiation a patient is exposed to, as well as having an impact on efficient usage of limited imaging resources.

The purpose of this study is to: a) evaluate radiologists’ inter-observer agreement of the quality of a history provided for ED abdominal CT scans and b) investigate if there is a correlation between the quality of history and number of imaging protocols that would be ordered by a radiologist.

## Materials and methods

### Patient selection

This retrospective study was approved by our tertiary care Research Institute Research Ethics Board (REB # 20140001-01H). Due to the retrospective nature of the study, written or oral consent by patients was waived by the Research Ethics Board. All data was anonymized and stored according to the explicitly stated REB rules which also follow the Helsinki Declaration. A retrospective chart review was performed of consecutive patients presenting to a tertiary care ED between September 1, 2012 and October 12, 2012: This was a period of time when no major construction or procedural changes were occurring in the ED or radiology departments and all documents were paper based in the ED. The teaching hospital is a 950 bed facility divided over three campuses, with two EDs as well as 166,000 emergency visits per year. The patients for this study were enrolled from a single campus which, in addition to providing generalized emergency care, serves as the regional cancer center. All patients who received an abdominopelvic CT at presentation were included. Patients who had more than one cross-sectional imaging study were included in the study as separate entries. Six trauma cases were excluded since in cases of trauma, history is often necessarily minimal and in cases of trauma, the radiologist or resident is always present at the CT console to determine the need for additional phases of imaging based on the imaging appearance of the initial images obtained.

All examinations were requested at the discretion of the ED physician, based on clinical concern. As per standard practice in our hospital, the resident or attending radiologist, who would be interpreting the examination, chose from a list of approximately 16 available protocols at the time of the requested CT scan based on their best judgment and consistent with appropriateness criteria followed by our tertiary care teaching institution (S1 Appendix). These protocols are listed in our computer order entry system, and there are corresponding CT protocols created on the CT scanner consoles for the technologists to choose. Some of the provided protocols are less commonly used in the ED, but may nevertheless be chosen in specific circumstances, and thus were provided as options for the purpose of this study (S2 Appendix). None of the requests were declined in this study. CT examinations were performed on either the GE HD750 Discovery 64-slice (Milwaukee, USA) or Toshiba Aquilion 64-slice multi detector CT scanners (Toshiba Medical Systems, Japan).

### Data retrieval

Requisition clinical history was extracted from the McKesson PACS HMI version 11.6 system (McKesson Corporation, Richmond, BC, Canada). During the time of this study, emergency requisitions for imaging were hand written, faxed to the technologist and protocolled directly by the resident or attending radiologist upon receiving the faxed requisition. Direct phone consultation with the radiologist was at the discretion of the emergency physician. During the ED visit, if the patient had an abdominal ultrasound prior to the CT, and the initial requisition contained a more in depth clinical presentation, this information was also collected and included in the provided history, since it represents the normal work flow in our department.

### Study design and data analysis

A 5 point Likert scale with 4 main components to analyze imaging requisition clinical history quality was created by a fellowship trained abdominal radiologist (AK) and a post-graduate radiology resident year 4 (PS). The key components in this scale mirror what is expected to be included in a consultation request to any other subspecialty service (including internal medicine and surgery) as described on the Canadian Medical Protective Agency website [13]. This includes a presenting complaint, past medical history or symptom evolution, objective laboratory or prior examination results and a differential diagnosis based on clinical evaluation. A graded scale was then developed using these criteria from 1–5, adding 1 point to the score for each major component that was present in the history. Requisitions containing only “abdominal pain” as the provided history or only a differential diagnosis that was too broad to be useful were given a score of 1 (see S1 Appendix for additional details).

Four additional radiologists, 2 abdominal imaging attendings with 8 (MM) and 7 (MEK) years of experience respectively) and 2 residents (post-graduate year 3 (CVP) and 4 (AJ)), were recruited to participate in the study. They participated in an introductory meeting which explained the grading classification developed for this study and they underwent a training session where 10 fictitious sample histories were reviewed and graded as a group (S1 Appendix).

Subsequently, as part of the study, reviewers were blinded to any other clinical information as well as to the CT itself. Although some clinical data may be present or available to the radiologist ultimately choosing the protocol, apart from a creatinine level, generally, other acute laboratory data values are not readily accessible when a protocol is chosen, given time constraints and particular work-flow in the emergency department at our teaching institution. For the purpose of this study, the 4 blinded reviewers were provided with patient age, sex and creatinine level. They reviewed the anonymized patient histories and were asked to provide a numerical history grade (1–5), and select a protocol from 16 commonly available abdomen/pelvic CT protocols used in the ED setting at our institution. An option for adding additional tailored protocols, or changing the examination to another modality (e.g. ultrasound or MRI) was given.

Finally, reviewers were asked to state how many protocols they felt could be reasonably used to evaluate the patient based on the history provided. Twenty one different protocol choices with regards to CT abdomen and pelvis were given to reviewers. (Protocol choices are listed in S1 Appendix).

### Statistical analysis

Inter-observer agreement was calculated between reviewers regarding A) Grade of the quality of history, B) Total number of protocols felt to be appropriate based on the provided history and C) Frequencies of protocols chosen by each reviewer (grouped into unenhanced, intravenous enhanced, multiphasic, and other modality (not CT)). Other modalities included ultrasound and MRI.

These results were expressed using intra-class correlation coefficients (ICC). ICC was chosen as it allows for correlation analysis of ordinal variables for a group of reviewers. Correlation between history grade and number of appropriate protocols was evaluated by means of Spearman’s rank correlation coefficient.

## Results

Histories provided on emergency requisitions for abdominal/pelvic CT on 350 consecutive patients were collected. The patient population average and median age was 58.4 years and 57 years (range, 15–97 years) with 166 male and 184 female patients included in the analysis. Eight patients received multiple CT examinations and were treated as individual entries given that the clinical history was different between the separate examinations.

**Fig 1:**
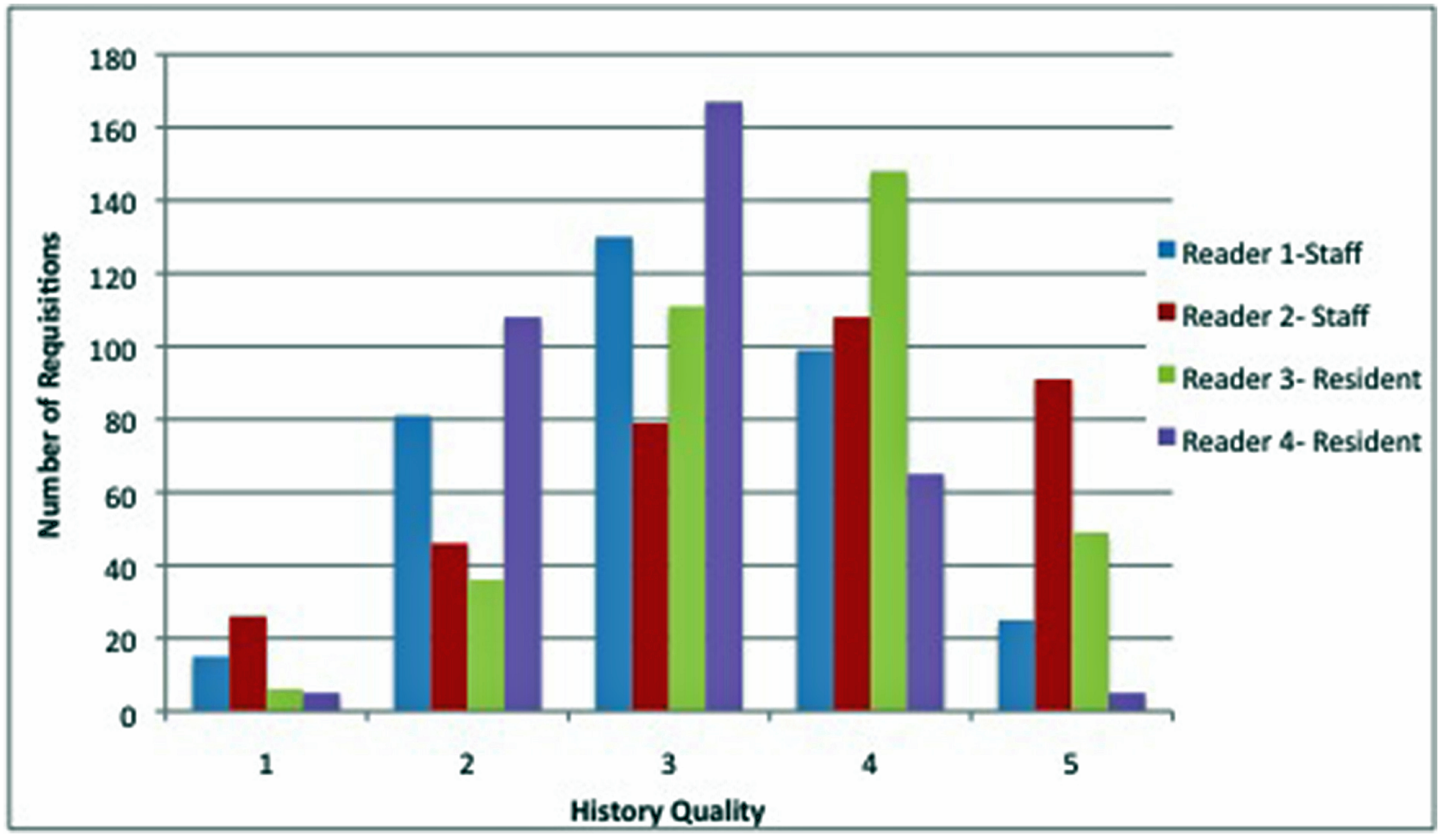
Chart representing the distribution of evaluated requisition based on history quality as evaluated by each individual reader. History Quality grades correspond to: 1 = None/ minimal, 2 = Some history, 3 = Moderate history, 4 = Good history, 5 = Excellent history.

Overall, no statistically significant difference in agreement for history grading was seen between staff and residents. Most of the provided histories were graded between 3 and 4 (Fig 1). A negative correlation was observed between history quality and number of chosen “appropriate” protocols between most reviewers. Lastly, the proportion of the type of protocols chosen does show similarities between the 4 reviewers.

When using the outlined history grading guidelines, there was substantial agreement amongst all 4 reviewers regarding history quality using the pilot grading scale (ICC 0.61, 0.48–0.71). Agreement amongst attending radiologists was substantial (ICC 0.69, 0.48–0.80). Agreement amongst residents was variable and ranged from no agreement to moderate agreement (ICC 0.55, -0.01–0.78).

**Fig 2:**
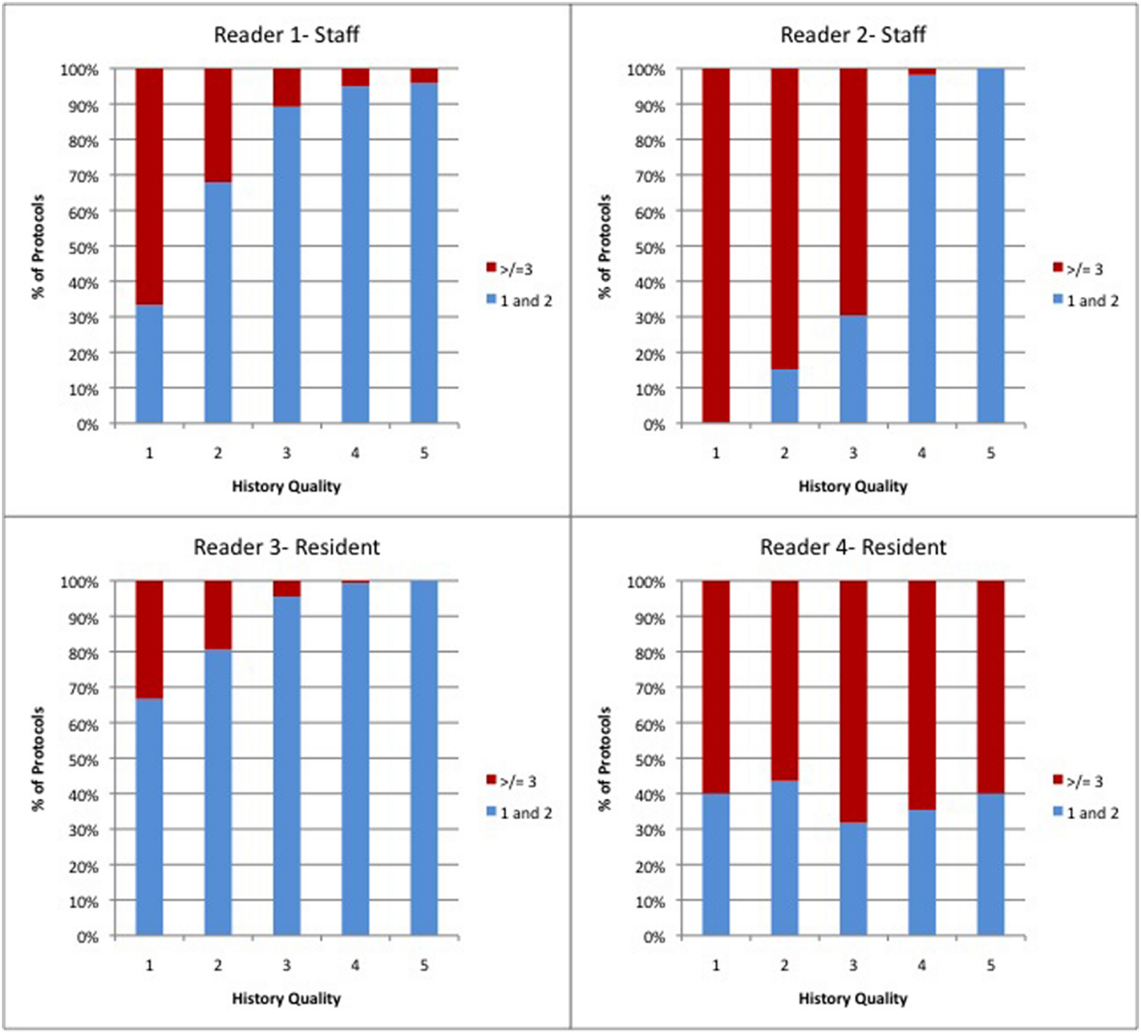
Graphic representation of observed negative correlation between history grade and number of protocols deemed appropriate to answer clinical question. The number of protocols considered was grouped into 2 groups (1 and 2 = blue, 3 or more = red). The number of protocols deemed appropriate (Percentage) in each history grade category is depicted. History Quality grades correspond to: 1 = None /minimal, 2 = Some history, 3 = Moderate history, 4 = Good history, 5 = Excellent history. Note: For each individual and each quality category, the overall number of protocols chosen was converted to a percentage and normalized to 100% (of their own choices).

The specific number of protocols deemed appropriate to answer the presenting clinical question showed considerable variability between reviewers. Specifically, there was poor agreement among the four reviewers with an ICC of 0.08 (-0.03–0.13). Of note, the agreement was better between attendings than residents (ICC 0.21 vs 0.05). A negative correlation was observed between history quality and number of chosen “appropriate” protocols in 3 of 4 reviewers (Spearman’s rho: -0.394, -0.639, -0.864, p <0.0001 for each reviewer). For the 4^th^ reviewer, there was no correlation observed between history quality and the number of chosen protocols (Spearman’s rho: 0.072, p = 0.179). The higher the quality of a history, the less potential protocols were chosen to answer the clinical question. This correlation was statistically significant in both attendings (rho: -0.763, 95% CI -0.793 to -0.731, p<0.0001) and residents (rho: -0.418, 95% CI -0.477 to -0.355, p <0.0001), however was stronger for attendings than residents (p<0.0001). (Fig 2)

There was a statistically significant difference between the type of imaging protocol chosen (Unenhanced, Intravenous enhanced, Multiphasic, Other/Non-CT study) among the 4 reviewers (ICC 0.99, 0.93–0.99, p<0.0001). This difference was primarily the result of variable utilization of multiphasic examinations and alternative examinations (e.g. ultrasound or MRI etc.). Intravenous enhanced single phase examinations made up the bulk of protocols chosen by all reviewers ranging between 54.6 and 62.9%. (Table 1). Reviewers decided to change the initial CT request to either Ultrasound or MRI in 3.1–19.4% of cases (Table 1).

**Table 1:** Distribution of protocols chosen by each reader based on the history provided.

	Attending 1	Attending 2	Resident 1	Resident 2
Intravenous enhanced	191/350 (54.6%)	201/350 (57.4%)	220/350 (62.8%)	214/350 (61.1%)
Unenhanced	69/350 (19.7%)	78/350 (22.3%)	72/350 (20.6%)	71/350 (20.3%)
Multiphase	22/350 (6.3%)	60/350 (17.1%)	27/350 (7.7%)	28/350 (8.0%)
Non-CT (US/MRI, other)	68/350 (19.4%)	11/350 (3.2%)	31/350 (8.9%)	37/350 (10.6%)

## Discussion

In this study, we attempted to quantitatively evaluate ED requisition histories provided and investigate the potential role they play in the decision-making process of the radiologist. We found a negative correlation between quality of a history provided and the number of protocols felt to be appropriate by the radiologist. This may have an impact on other aspects of medical imaging and highlights the importance of communication between specialties as well as a need for standardization of this initial step in patient imaging care [13].

It is not expected that clinicians will provide an exhaustive history, but rather, key points which may help radiologists choose imaging as effectively as possible. With increasing access to electronic medical records, radiologists may have increasing access to patient history, however, the acute clinical interaction at the time of the ED visit may still provide additional information required by the radiologist [11, 12].

Standardization is a goal of quality imaging in radiology. This includes use of standardized template reporting and use of homogeneous technical parameters related to acquisition of images [14–16]. The steps related to choosing the imaging modality and the protocol within a particular modality (CT and MRI in particular) can have a significant impact on the ability of a radiologist to make a particular diagnosis which is why this study was undertaken.

The reviewers in our study showed substantial agreement when evaluating history quality according to the outlined grading system. With regards to choosing an appropriate protocol, there was marked variability between reviewers. Reviewers predominantly chose the single phase, “contrast-enhanced CT” acquired in the portal venous phase, without oral/enteric contrast protocol. This is generally the most commonly used protocol at our institution since it is fast to perform, allows for a good global assessment of the abdomen and is associated with only moderate radiation exposure, compared to other available protocols. The age and sex of the patient was provided to the radiologists and thus, it was expected that the choice of exam protocols would be affected by this information, with more radiation sensitive individuals (generally < 30 years of age) being offered imaging that does not include ionizing radiation (such as ultrasound or MRI). There was an observed negative correlation between history quality and number of protocols deemed appropriate to answer the clinical question; despite a high variability between reviewers in the overall number of protocols considered adequate to address the chief complaint. This effect was present in 3 of the 4 reviewers, with a stronger correlation among attending radiologists.

The variability in number of considered protocols may be partially accounted for by the broad differential diagnosis for many ED presenting complaints. The number of protocols chosen may also be confounded and limited by the individuality of each radiologist and their specific practice style. Even in the era of evidence-based medicine, there are many unanswered questions and thus, the “art of medicine” continues to play a role in the practice of radiology. This position can be supported by the persistent variability in reporting practices within avenues of radiology such as the terminology in describing, as well as management of incidental thyroid nodules [17, 18]. Greater inter-observer variability in the resident reviewers may reflect the experience level of the reader or familiarity with the presenting problem, though further research is needed to fully elucidate the reasons.

Some of the findings in the current study also highlight the need for development of standards of expectations for radiologists and how this information may lead to more consistent algorithms for choosing appropriate imaging. This is important to ensure radiology’s continued value-added role in patients’ overall health care as well as in the development of Clinical Decision Support tools in the future.

In order to perform the best possible imaging for patients, an adequate clinical history is required [19, 20]: A meta-analysis of 16 studies including 1 on CT published in JAMA documented that a test’s accuracy is improved when interpreted with an accurate clinical history [8]. Requisitions that are too brief, incomplete, illegible and in rare cases, misleading, continue to be a problem that can lead to a breakdown of communication between the radiologist and referring physician [14, 21 22, 23]. As a result, potential medical errors such as performing the wrong imaging study or choosing a protocol that cannot answer the question, still occur [23]. Improving the quality of history can result in fewer considered protocols by the radiologist. What constitutes a “high quality” requisition has not been completely elucidated. We acknowledge that history quality is subjective and there are many criteria that can be included under the umbrella of quality. This subjectivity is a limitation of our current study. However, at present, there are no available gold standards for determining history quality and different authors have used different techniques and statistical analyses to evaluate quality of history in a few previously published manuscripts [11, 12]. As the imaging requisition is a medical document included in the patient encounter, the grading system utilized in this study was based on criteria described in guidelines for documentation of patient encounters and subsequent papers regarding the guidelines’ clinical application [24, 25].

A possible limitation of this study is the patient population chosen for this study. In the emergency department, the complexity of cases can vary significantly, but often, patients present with one particular ailment (e.g. appendicitis, cholecystitis, renal calculi) and thus the importance of the provided history may not be as evident in this group of patients. However, the 5 point grading system used for this project could be transferred to other patient populations and tested in other aspects of healthcare in the future (e.g. oncology follow-up). Other studies including the one by Pevnick used a 3 point scale; our 5 point scale allowed a wider range of grading to be more specific in terms of definitions and requirements. Evaluation of number of protocols and indication quality for each case is also another possible study limitation. In this study, reviewers simultaneously rated the number of protocols and indication quality for each case which ideally should have been performed by separate reviewers or at different points in time. However, this methodology is reflective of daily practise where a single radiologist would unconsciously judge the quality of a requisition history and subsequently determine the appropriate protocol(s) shortly after.

As computer based order entry systems become more prevalent, including clinical decision support tools and as radiology information systems (RIS) systems become integrated into hospital PACS, the ease of obtaining clinical knowledge may also improve [26]. Nevertheless, the importance of initial communication between ED physicians and radiologists should be fostered and standardized to ensure that all patients receive the best possible care, using the least resources possible. In busy clinical practices, with increasing focus on radiology report turn-around time, the ability of radiologists to track down physicians making requests becomes more challenging. The substantial agreement observed in this pilot study, with regards to history quality, shows that there can be a more structured approach to presenting and evaluating requisition histories. Further research evaluating which components of requesting history provide the most useful information for radiologists would enable more efficient implementation in daily practice. Electronic guidance during the order entry process can also act as safeguard to ensure appropriate information is included and a high quality requesting history is provided [27]. We have shown that with a higher quality history provided on a requisition, less protocols are considered by the protocolling radiologist.

## Conclusion

With regards to CT in the ED, we have found a negative correlation between quality of a provided history and the number of protocols identified as potentially appropriate by the radiologist choosing the imaging protocol. Despite variability in interrater reliability, a smaller number of protocols were deemed appropriate to answer the clinical question as the quality of the provided improved. Finally, our findings suggest that history quality can be assessed by more objective criteria than simply by subjective personal evaluation.

## Supporting information

S1 Appendix Additional information regarding the proposed grading system, the criteria used and the training provided to the radiologists and radiology residents prior to participating in the study. (DOC)

S2 Appendix Detailed information regarding each type of Abdominal/pelvic CT available for radiologists to use in the emergency department (programmed into the CT consoles for technologists to use). Of note, changing a protocol from CT to MRI or ultrasound or “other” are not true CT protocols. Also, we excluded trauma cases, therefore the “trauma CT” protocol is not relevant to this study. Finally the “CT enterography chronic low grade small bowel obstruction” protocol is never used in the emergency setting and is not even programmed into the ER CT scanner. Therefore of the 21 options listed, only 16 CT protocols were actually available as discrete CT protocols.
(DOC)

S1 Data Raw Data from History by 4 blinded reviewers. (XLS)
