# Supplementary material for: Impact of clinical history on choice of abdominal/pelvic CT protocol in the Emergency Department
Source: PLoS One. 2018 Aug 7;13(8):e0201694. doi: 10.1371/journal.pone.0201694 (PMC6080782; doi:10.1371/journal.pone.0201694)
Supplement: S1 Appendix — (DOC) [file pone.0201694.s001.doc]

**Appendix**

**Grading System**

The developed grading system to evaluate the assessed requisition histories was modeled on the clinical encounter literature and guidelines for documentation, as no established way of quantifying a requisition is available. The key components included mirror what is expected to be included in a consultation request to any other subspecialty service (including internal medicine and surgery). The criteria included were believed to encompass key questions or pieces of the clinical presentation and past medical history that a radiologist would want in inquire about. The included criteria are as follows:

**Criteria:**

- Presenting complaint

- Past medical/surgical history or descriptor of symptom evolution during presentation

- Objective laboratory or prior examination results. This includes verbal descriptions – i.e. fever, hematuria.

- Differential diagnosis based on clinical assessment

A graded scale was then developed using these criteria from 1-5, adding 1 point to the score for each major component that was present in the history. Requisitions containing only “abdominal pain” as the provided history or only a differential diagnosis that was too broad to be useful were given a score of 1.

**Grading System**

Grade 1

- No/Minimal history
- One word history
- Differentials that are too broad or give you no guiding information
- Ex:
  - Abdominal Pain

Grade 2

- Some history (1 criterion)
- Differential
  - Histories with poor description of presenting problem and a top differential diagnosis were included in this Grade.
- Ex:
  - Pain, RO Crohn’s
  - RO Appendicitis

Grade 3

- Moderate history (2 criteria)
- Ex:
  - History of recent surgery RO appendicitis
  - Diffuse abdominal pain and elevated white count
  - Nausea and repetitive vomiting, RO small bowel obstruction

Grade 4

- Good History (3 criteria)
- Ex:
  - Well 2 days prior, acute onset sharp LLQ pain that has been progressive, US negative for torsion
  - Post partum with RLQ pain, WBC 11.2
  - Vasculopath with cool L great toe ? ischemic toe
  - L flank pain with hematuria RO stones

Grade 5 – Excellent history (4 criteria)

- Ex:
  - Intense abdominal pain, hypotensive with known Hx of AAA, RO rupture
  - Sudden onset L flank pain with hematuria, RO stones

**Training**

A training session with complete description of the criteria and scale grades was done with the readers. They all then evaluated the following 10 histories prior to evaluating the patient histories. The readers’ evaluation of the test histories was reviewed, following which the results were discussion with the reader and individual feedback regarding the use of the grading system was given to ensure a uniform adaptation of the grading system.

**Test Histories**

| **Test #** | **Gender** | **Age** | **Cr** | **Clinical History** | **Quality of History** |
| --- | --- | --- | --- | --- | --- |
| 1 | M | 30 | 60 | RO Appendicitis | 2 |
| 2 | M | 46 | 45 | Hematemesis, EtOH abuse, RO varices | 4 |
| 3 | F | 54 | 48 | Abdominal pain, vomiting, nausea | 2 |
| 4 | F | 13 | 22 | RO Diverticulitis, ischemic bowel, abdominal hernia | 1 |
| 5 | F | 24 | 60 | Hx of Crohns, increasing abdominal pain, RO acute inflammation or abscess | 4 |
| 6 | M | 87 | 90 | Renal colic | 2 |
| 7 | M | 69 | 85 | Abdominal pain | 1 |
| 8 | M | 55 | 72 | Whipples, not tolerating PO, fever RO stricture | 5 |
| 9 | F | 48 | 34 | Swallowed Nail, abdominal pain. RO perforation | 3 |
| 10 | M | 39 | 140 | Elevated Cr with R flank pain, previously healthy, RO colic/urinary obstruction | 5 |

**Protocols:**

Common Utilized Body CT Protocols allowed for selection for provided histories included:

1) CT abdomen and pelvis (non-contrast)

2) CT Abdomen and Pelvis (non-contrast but with positive oral contrast)

3) Renal colic (non-contrast, low dose, from top of kidneys to base of bladder)

4) CTAP + C

5) CTAP + C + oral +/- rectal

6) Aortic dissection

7) Appendix AP+C

8) Pancreatitis -/+

9) Pancreatic mass protocol (-/+C (3 phase)

10) Triphasic liver

11) Bariatric protocol (CTAP+C + long + 9% on table)

12) CT Enterography

13) Ischemia protocol

14) CT Hematuria -/+

15) CT Cystogram

16) CT Trauma

17) CT Enterography Anemia assessment

18) CT enterography chronic low grade small bowel obstruction

19) Other

20) Change study to US

21) Change study to MRI
