# Supplementary material for: Impact of clinical history on choice of abdominal/pelvic CT protocol in the Emergency Department
Source: PLoS One. 2018 Aug 7;13(8):e0201694. doi: 10.1371/journal.pone.0201694 (PMC6080782; doi:10.1371/journal.pone.0201694)
Supplement: S2 Appendix — Of note, changing a protocol from CT to MRI or ultrasound or “other” are not true CT protocols. Also, we excluded trauma cases, therefore the “trauma CT” protocol is not relevant to this study. Finally the “CT enterography chronic low grade small bowel obstruction” protocol is never used in the emergency setting and is not even programmed into the ER CT scanner. Therefore of the 21 options listed, only 16 CT protocols were actually available as discrete CT protocols. (DOC) [file pone.0201694.s002.doc]

LIST OF ADBOMEN/PELVIS CT PROTOCOLS USED IN THE ED

# ABDOMEN PELVIS WITH INTRAVENOUS CONTRAST

| **Clinical Indication** | ED PATIENTS WITH PAIN NYD,POST OP EVALUATION,SEARCH FOR PRIMARY CA OR IF CONTRAINDICATION TO ORAL CONTRAST | | | | | | | |
| --- | --- | --- | --- | --- | --- | --- | --- | --- |
| **Protocol Code** | **AP + ©** | | | | | | | |
| **Contrast** | **ORAL CONTRAST** – 2% Diatrizoate Meglumine Long, 2X600ml, 1/2cup at O min, 30 min, 60 min, 90 min, Make sure to have some on the table to fill stomach | | | | | | | |
|  | **I.V. CONTRAST** – 80 –100ml Iopamidol 370, unless alternate dose specified by Radiologist. | | | | | | | |
| **CT Technique**  **Without IV Contrast** |  | | | | | | | |
|  | kV | mA | Scan type  Rotation time | Scan  Thickness  (mm) | Scan mode | Speed  (mm) | Recon thickness  (mm) | Algo |
|  |  |  |  |  |  |  |  |  |
| **CT Technique**  **With IV Contrast** | PVP-Abdomen and pelvis – 5mm - 65 sec delay 2 ml/sec  Use Auto mA if possible for patient under 250 lbs.  NOISE INDEX 41.4 | | | | | | | |
|  | kV | mA  **ASiR 40%** | Scan type  Rotation time | Scan  Thickness  (mm) | Scan mode | Speed  (mm) | Recon thickness  (mm) | Algo |
|  | 120 | 100-450 | HELICAL .8 | 1.25 | 0.984:1 | 39.37 | 5 AXIAL  2 CORONAL | STANDARD |
|  |  |  |  |  |  |  |  |  |
|  | kV | mA | Scan type  Rotation time | Scan  Thickness  (mm) | Scan mode | Speed  (mm) | Recon thickness  (mm) | Algo |
|  |  |  |  |  |  |  |  |  |
| **Comment** | The default for patients < 35 years, and < 130 lbs would be 100KV. | | | | | | | |

# ABDOMEN PELVIS WITH INTRAVENOUS CONTRAST AND LONG DRINKS

| **Clinical Indication** | ED PATIENTS/INPATIENTS WITH SUSPECTED POST-OP ABSCESS.OUTPATIENTS WITH PAIN, LYMPHOMA-STAGING /RESTAGING; COLORECTAL CA-STAGING/FOLLOW UP; PANCREAS CA FOLLOW UP; GE JUNCTION/GASTRIC CA FOLLOW UP, LIVER LESION FOLLOW UP, RCC/TCC AND BLADDER CA FOLLOW UP; GYNE CANCER STAGING/FOLLOW UP, SEMINOMA STAGING/RESTAGING, PROSTATE STAGING/RESTAGING; FEBRILE NEUTROPENIA | | | | | | | |
| --- | --- | --- | --- | --- | --- | --- | --- | --- |
| **Protocol Code** | **AP + © LONG** | | | | | | | |
| **Contrast** | **ORAL CONTRAST** – 2% Diatrizoate Meglumine Long, 2X600ml, 1/2cup at O min, 30 min, 60 min, 90 min, Make sure to have some on the table to fill stomach | | | | | | | |
|  | **I.V. CONTRAST** – 80 –100ml Iopamidol 370, unless alternate dose specified by Radiologist. | | | | | | | |
| **CT Technique**  **Without IV Contrast** |  | | | | | | | |
|  | kV | mA | Scan type  Rotation time | Scan  Thickness  (mm) | Scan mode | Speed  (mm) | Recon thickness  (mm) | Algo |
|  |  |  |  |  |  |  |  |  |
| **CT Technique**  **With IV Contrast** | PVP-Abdomen and pelvis – 5mm - 65 sec delay 2 ml/sec  Use Auto mA if possible for patient under 250 lbs.  NOISE INDEX 41.4 | | | | | | | |
|  | kV | mA  **ASiR 40%** | Scan type  Rotation time | Scan  Thickness  (mm) | Scan mode | Speed  (mm) | Recon thickness  (mm) | Algo |
|  | 120 | 100-450 | HELICAL .8 | 1.25 | 0.984:1 | 39.37 | 5 AXIAL  2 CORONAL | STANDARD |
|  |  |  |  |  |  |  |  |  |
|  | kV | mA | Scan type  Rotation time | Scan  Thickness  (mm) | Scan mode | Speed  (mm) | Recon thickness  (mm) | Algo |
|  |  |  |  |  |  |  |  |  |
| **Comment** | The default for patients < 35 years, and < 130 lbs would be 100KV. | | | | | | | |

ABDOMEN PELVIS, NO IV CONTRAST, NO DRINKS

| **Clinical Indication** | AAA FOR SIZE, ED PATIENT WHO CANNOT HAVE IV CONTRAST AGENT DUE TO LOW CREATININE CLEARANCE | | | | | | | |
| --- | --- | --- | --- | --- | --- | --- | --- | --- |
| **Protocol Code** | **AP -** | | | | | | | |
| **Contrast** | **ORAL CONTRAST** – 2% Diatrizoate Meglumine Long, 2X600ml, 1/2cup at O min, 30 min, 60 min, 90 min, Make sure to have some on the table to fill stomach | | | | | | | |
|  | **I.V. CONTRAST –** No I.V. Contrast | | | | | | | |
| **CT Technique**  **Without IV Contrast** | Abdomen and Pelvis – Without – 5mm  Use Auto mA if possible for patient under 250 lbs.  NOISE INDEX 32 | | | | | | | |
|  | kV | mA  **ASiR 40%** | Scan type  Rotation time | Scan  Thickness  (mm) | Scan mode | Speed  (mm) | Recon thickness  (mm) | Algo |
|  | 120 | 100-400 | HELCIAL .8 | 5 | 0.984:1 | 39.37 | 5 | STANDARD |
| **CT Technique**  **With IV Contrast** |  | | | | | | | |
|  | kV | mA | Scan type  Rotation time | Scan  Thickness  (mm) | Scan mode | Speed  (mm) | Recon thickness  (mm) | Algo |
|  |  |  |  |  |  |  |  |  |
|  |  |  |  |  |  |  |  |  |
|  | kV | mA | Scan type  Rotation time | Scan  Thickness  (mm) | Scan mode | Speed  (mm) | Recon thickness  (mm) | Algo |
|  |  |  |  |  |  |  |  |  |
| **Comment** |  | | | | | | | |

ABDOMEN PELVIS WITH LONG DRINKS, NO IV CONTRAST

| **Clinical Indication** | AAA FOR SIZE, ED PATIENT WHO CANNOT HAVE IV CONTRAST AGENT DUE TO LOW CREATININE CLEARANCE | | | | | | | |
| --- | --- | --- | --- | --- | --- | --- | --- | --- |
| **Protocol Code** | **AP -** | | | | | | | |
| **Contrast** | **ORAL CONTRAST –** No Drinks | | | | | | | |
|  | **I.V. CONTRAST –** No I.V. Contrast | | | | | | | |
| **CT Technique**  **Without IV Contrast** | Abdomen and Pelvis – Without – 5mm  Use Auto mA if possible for patient under 250 lbs.  NOISE INDEX 32 | | | | | | | |
|  | kV | mA  **ASiR 40%** | Scan type  Rotation time | Scan  Thickness  (mm) | Scan mode | Speed  (mm) | Recon thickness  (mm) | Algo |
|  | 120 | 100-400 | HELCIAL .8 | 5 | 0.984:1 | 39.37 | 5 | STANDARD |
| **CT Technique**  **With IV Contrast** |  | | | | | | | |
|  | kV | mA | Scan type  Rotation time | Scan  Thickness  (mm) | Scan mode | Speed  (mm) | Recon thickness  (mm) | Algo |
|  |  |  |  |  |  |  |  |  |
|  |  |  |  |  |  |  |  |  |
|  | kV | mA | Scan type  Rotation time | Scan  Thickness  (mm) | Scan mode | Speed  (mm) | Recon thickness  (mm) | Algo |
|  |  |  |  |  |  |  |  |  |
| **Comment** |  | | | | | | | |

TRAUMA WITH IV CONTRAST

| **Clinical Indication** | TRAUMA | | | | | | | |
| --- | --- | --- | --- | --- | --- | --- | --- | --- |
| **Protocol Code** | **CHEST ABDOMEN/PELVIS Trauma Protocol** | | | | | | | |
| **Contrast** | **ORAL CONTRAST –** None | | | | | | | |
|  | I.V. CONTRAST - 115 Iopamidol, unless alternate dose specified by Radiologist.75 ml contrast @2.5ml/sec 40ml saline @ 2.0ml then injector pause of 15 seconds 40ml of contrast and 20 ml of saline @3.2 /sec90 second scan delay | | | | | | | |
| **CT Technique**  **Without IV Contrast** |  | | | | | | | |
|  | kV | mA | Scan type  Rotation time | Scan  Thickness  (mm) | Scan mode | Speed  (mm) | Recon  Thickness  (mm) | Algo |
|  |  |  |  |  |  |  |  |  |
| **CT Technique**  **With IV Contrast** | CHEST ABDOMEN PELVIS  Use Auto mA if possible for patient under 250 lbs.  NOISE INDEX 41.4 | | | | | | | |
|  | kV | mA | Scan type  Rotation time | Scan  Thickness  (mm) | Scan mode | Speed  (mm) | Recon thickness  (mm) | Algo |
|  | 120 | 100-500 | HELICAL .6 | 0.625 | 0.984:1 | 39.37 | 2 AXIAL  2 CORONAL  2 SAGITTAL | STANDARD |
|  |  |  |  |  |  |  |  |  |
|  |  |  |  |  |  | LUNG | Recon 2 thickness  2.5mm | Algo  LUNG |
|  |  |  |  |  |  | SPINE  19cm FOV | Recon 3 thickness  1.25mm | Algo  Bone plus |
| **Comment** | Scan Chest, Abdomen and Pelvis together in one scan  Create 2mm Sagittal and Coronal images of spine and send to PACS  Axial spine does not go to PACS | | | | | | | |

**CHEST, ABDOMEN/PELVIS DISSECTION PROTCOL**

| **Clinical Indication** | SUSPECTED AORTIC DISSECTION (THORACIC OR ABDOMINAL) | | | | | | | |
| --- | --- | --- | --- | --- | --- | --- | --- | --- |
| **Protocol Code** | **DISSECTION PROTCOL** | | | | | | | |
| **Contrast** | **ORAL CONTRAST –** None | | | | | | | |
|  | I.V. CONTRAST - 115 Iopamidol, unless alternate dose specified by Radiologist.75 ml contrast @2.5ml/sec 40ml saline @ 2.0ml then injector pause of 15 seconds 40ml of contrast and 20 ml of saline @3.2 /sec90 second scan delay | | | | | | | |
| **CT Technique**  **Without IV Contrast** | Abdomen and Pelvis – Without – 5mm  Use Auto mA if possible for patient under 250 lbs.  NOISE INDEX 32 | | | | | | | |
|  | kV | mA | Scan type  Rotation time | Scan  Thickness  (mm) | Scan mode | Speed  (mm) | Recon  Thickness  (mm) | Algo |
|  | 120 | 100-400 | HELCIAL .8 | 5 | 0.984:1 | 39.37 | 5 | STANDARD |
| **CT Technique**  **With IV Contrast** | CHEST ABDOMEN PELVIS  Use Auto mA if possible for patient under 250 lbs.  NOISE INDEX 41.4 | | | | | | | |
|  | kV | mA | Scan type  Rotation time | Scan  Thickness  (mm) | Scan mode | Speed  (mm) | Recon thickness  (mm) | Algo |
|  | 120 | 100-500 | HELICAL .6 | 0.625 | 0.984:1 | 39.37 | 2 AXIAL  2 CORONAL  2 SAGITTAL | STANDARD |
|  |  |  |  |  |  |  |  |  |
|  |  |  |  |  |  | LUNG | Recon 2 thickness  2.5mm | Algo  LUNG |
| **Comment** | Scan Chest, Abdomen and Pelvis together in one scan  Create 2mm Sagittal and Coronal images of spine and send to PACS  Axial spine does not go to PACS | | | | | | | |

MULTI PHASE LIVER WITH INTRAVENOUS CONTRAST

| **Clinical Indication** | LIVER LESION CHARACTERIZATION | | | | | | | |
| --- | --- | --- | --- | --- | --- | --- | --- | --- |
| **Protocol Code** | **LIVER LESION + ©** | | | | | | | |
| **Contrast** | **ORAL CONTRAST** – No Drinks | | | | | | | |
|  | **I.V. CONTRAST –** 90 –110ml Iopamidol 370, unless alternate dose specified by Radiologist. | | | | | | | |
| **CT Technique**  **Without IV Contrast** | Abdomen Without – 5mm (if chosen by Radiologist)  Use Auto mA if possible for patient under 250 lbs.  NOISE INDEX 30 | | | | | | | |
|  | kV | mA  **ASiR 40%** | Scan type  Rotation time | Scan  Thickness  (mm) | Scan mode | Speed  (mm) | Recon thickness  (mm) | Algo |
|  | 120 | 100-400 | HELCIAL .8 | 5 | 0.984:1 | 39.37 | 5 | STANDARD |
|  | LIVER- 2.5mm  Arterial Phase - BOLUS TRACKING- from the Abd Aorta - 20 sec from 100 HU  3.2 cc /sec – Iopamidol 370  Use Auto mA if possible for patient under 250 lbs.  NOISE INDEX 41.4 | | | | | | | |
|  | kV | mA  **ASiR 40%** | Scan type  Rotation time | Scan  Thickness  (mm) | Scan mode | Speed  (mm) | Recon thickness  (mm) | Algo |
|  | 120 | 100-500 | HELCIAL .8 | 0.625 | 0.984:1 | 39.37 | 2 AXIAL  2 CORONAL | STANDARD |
|  | PVP- Liver – 5mm - 50 sec from 100 HU  Pelvis – PVP 5mm (if chosen by Radiologist)  Delayed Liver – 5mm -300 sec from 100 HU (if chosen by Radiologist) | | | | | | | |
|  | kV | mA  **ASiR 40%** | Scan type  Rotation time | Scan  Thickness  (mm) | Scan mode | Speed  (mm) | Recon thickness  (mm) | Algo |
|  | 120 | 100-400 | HELCIAL .8 | 1.25 | 0.984:1 | 39.37 | 5 AXIAL | STANDARD |
| **Comment** | Doctor’s note-consider MRI for first line for all patients –esp. under 50 yrs with likely benign lesions  **Tech’s note** – This protocol is routinely done only as with contrast HAP, PVP Abdomen. Selection of add-on choices will be made by Radiologist | | | | | | | |

# SUSPECTED PANCREATIC CANCER WITH IV CONTRAST

| **Clinical Indication** | PANCREAS CANCER SUSPECTED OR FOR STAGING | | | | | | | |
| --- | --- | --- | --- | --- | --- | --- | --- | --- |
| **Protocol Code** | **PANCREATIC CA+©** | | | | | | | |
| **Contrast** | **ORAL CONTRAST –** H2O, 1X600ml  PATIENT DRINK THE ENTIRE CUP ON THE TABLE | | | | | | | |
|  | **I.V. CONTRAST –** 90 –110ml Iopamidol 370, unless alternate dose specified by Radiologist. | | | | | | | |
| **CT Technique**  **Without IV Contrast** | Without Pancreas - 2.5mm  Use Auto mA if possible for patient under 250 lbs.  NOISE INDEX 30 | | | | | | | |
|  | kV | mA  **ASiR 40%** | Scan type  Rotation time | Scan  Thickness  (mm) | Scan mode | Speed  (mm) | Recon thickness  (mm) | Algo |
|  | 120 | 100-450 | Helical .8 | 2.5 | 0.984:1 | 39.37 | 2.5 | STANDARD |
| **CT Technique**  **With IV Contrast** | Pancreas – Full Abdomen FOV 1.25 mm  Arterial Phase-BOLUS TRACKING-from the Abd aorta-15 sec from 100 HU  Use Auto mA if possible for patient under 250 lbs.  NOISE INDEX 41.4  3.2ml/sec | | | | | | | |
|  | kV | mA  **ASiR 40%** | Scan type  Rotation time | Scan  Thickness  (mm) | Scan mode | Speed  (mm) | Recon thickness  (mm) | Algo |
|  | 120 | 100-500 | HELCIAL .8 | 1.25 | 0.984:1 | 39.37 | 1 AXIAL  2 CORONAL | STANDARD |
|  | Abdomen and Pelvis-2.5 mm  PVP - BOLUS TRACKING -50 sec from 100HU | | | | | | | |
|  | kV | mA  **ASiR 40%** | Scan type  Rotation time | Scan  Thickness  (mm) | Scan mode | Speed  (mm) | Recon thickness  (mm) | Algo |
|  | 120 | 100-500 | HELCIAL .8 | 1.25 | 0.984:1 | 39.37 | 2 AXIAL  2 CORONAL | STANDARD |
| **Comment** |  | | | | | | | |

# PANCREATITIS WITH INTRAVENOUS CONTRAST

| **Clinical Indication** | PANCREATITIS, Common Bile Duct STONE, CHOLANGITIS | | | | | | | |
| --- | --- | --- | --- | --- | --- | --- | --- | --- |
| **Protocol Code** | **PANCREATITIS + ©** | | | | | | | |
| **Contrast** | **ORAL CONTRAST –** H2O, 1X600ml  PATIENT DRINK THE ENTIRE CUP ON THE TABLE | | | | | | | |
|  | **I.V. CONTRAST –** 80 –100ml Iopamidol 370, unless alternate dose specified by Radiologist. | | | | | | | |
| **CT Technique**  **Without IV Contrast** | Abdomen Without - 2.5mm  Use Auto mA if possible for patient under 250 lbs.  NOISE INDEX 30 | | | | | | | |
|  | kV | mA  **ASiR 40%** | Scan type  Rotation time | Scan  Thickness  (mm) | Scan mode | Speed  (mm) | Recon thickness  (mm) | Algo |
|  | 120 | 100-450 | HELCIAL .8 | 1.25 | 0.984:1 | 39.37 | 2.5 AXIAL  2 CORONAL | STANDARD |
| **CT Technique**  **With IV Contrast** | PVP Abdomen and Pelvis – 5mm - 65 sec delay 2ml/sec  NOISE INDEX 41.4 | | | | | | | |
|  | kV | mA  **ASiR 40%** | Scan type  Rotation time | Scan  Thickness  (mm) | Scan mode | Speed  (mm) | Recon thickness  (mm) | Algo |
|  | 120 | 100-450 | HELCIAL .8 | 1.25 | 0.984:1 | 39.37 | 2.5 AXIAL  2 CORONAL | STANDARD |
|  |  |  |  |  |  |  |  |  |
|  | kV | mA | Scan type  Rotation time | Scan  Thickness  (mm) | Scan mode | Speed  (mm) | Recon thickness  (mm) | Algo |
|  |  |  |  |  |  |  |  |  |
| **Comment** | **Doctor’s may omit pelvis by explicitly stating no pelvis | | | | | | | |

#

# RENAL COLIC

| **Clinical Indication** | RENAL COLIC, RENAL CALCULI, URETERIC CALCULI | | | | | | | |
| --- | --- | --- | --- | --- | --- | --- | --- | --- |
| **Protocol Code** | **RENAL COLIC** | | | | | | | |
| **Contrast** | **ORAL CONTRAST –** No Drinks | | | | | | | |
|  | **I.V. CONTRAST –** No I.V. Contrast | | | | | | | |
| **CT Technique**  **Without IV Contrast** | LOW DOSE Kidneys to Ischial Tuberosities - 2.5mm Always use auto mA min 25 – 200 max NOISE INDEX 40 | | | | | | | |
|  | kV | mA  **ASiR 40%** | Scan type  Rotation time | Scan  Thickness  (mm) | Scan mode | Speed  (mm) | Recon thickness  (mm) | Algo |
|  | 120 | 100-180 | Helical 0.5 | 2.5 | 0.984:1 | 39.37 | 2.5 | standard |
| **CT Technique**  **With IV Contrast** |  | | | | | | | |
|  | kV | mA | Scan type  Rotation time | Scan  Thickness  (mm) | Scan mode | Speed  (mm) | Recon thickness  (mm) | Algo |
|  |  |  |  |  |  |  |  |  |
|  |  |  |  |  |  |  |  |  |
|  | kV | mA | Scan type  Rotation time | Scan  Thickness  (mm) | Scan mode | Speed  (mm) | Recon thickness  (mm) | Algo |
|  |  |  |  |  |  |  |  |  |
| **Comment** | Low dose exam DLP should not exceed 300 mgy-cm | | | | | | | |

# RENAL MASS WITH INTRAVENOUS CONTRAST

| **Clinical Indication** | RENAL LESIONS (RCC, BOSNIAK CLASSIFICATION) | | | | | | | |
| --- | --- | --- | --- | --- | --- | --- | --- | --- |
| **Protocol Code** | **RENAL MASS** **+ ©** | | | | | | | |
| **Contrast** | **ORAL CONTRAST** – No Drinks | | | | | | | |
|  | **I.V. CONTRAST –** 90 –110ml Iopamidol 370, unless alternate dose specified by Radiologist. | | | | | | | |
| **CT Technique**  **Without IV Contrast** | Kidneys Without - 2.5mm  NOISE INDEX 41.4 | | | | | | | |
|  | kV | mA | Scan type  Rotation time | Scan  Thickness  (mm) | Scan mode | Speed  (mm) | Recon thickness  (mm) | Algo |
|  | 120 | 100-450 | Helical .8 | 1.25 | 0.984:1 | 39.37 | 2 AXIAL | STANDARD |
| **CT Technique**  **With IV Contrast** | Kidneys ONLY - 2.5mm  CMP (Cortical Medullary phase) - BOLUS TRACKING-15 sec from 100 HU  3.2 ml/sec  NOISE INDEX 41.4 | | | | | | | |
|  | kV | mA | Scan type  Rotation time | Scan  Thickness  (mm) | Scan mode | Speed  (mm) | Recon thickness  (mm) | Algo |
|  | 120 | 100-450 | HELCIAL .8 | 1.25 | 0.984:1 | 39.37 | 2 AXIAL  2 CORONAL | STANDARD |
|  | Nephrographic Phase-Abdomen - 2.5mm BOLUS TRACKING- 80 sec from 100 HU  Pelvis 2.5mm(if chosen by Radiologist) | | | | | | | |
|  | kV | mA | Scan type  Rotation time | Scan  Thickness  (mm) | Scan mode | Speed  (mm) | Recon thickness  (mm) | Algo |
| ABDOMEN | 120 | 100-450 | HELCIAL .8 | 1.25 | 0.984:1 | 39.37 | 2 AXIAL  2 CORONAL | STANDARD |
| **Comment** | ****Use same kV, mA, rotation speed, pitch, and noise index for W/O, CMP and NP phases. This is so reliable HU enhancement assessment can be made** | | | | | | | |

# HEMATURIA WITH INTRAVNEOUS CONTRAST

| **Clinical Indication** | HEMATURIA NYD AND CT UROGRAPHY INDICATIONS | | | | | | | |
| --- | --- | --- | --- | --- | --- | --- | --- | --- |
| **Protocol Code** | **HEMATURIA +** © | | | | | | | |
| **Contrast** | **ORAL CONTRAST –** H2O, 1X600ml 30 min prior to scan OR  If Patient unable to drink 250cc IV NS bolus before scan (requires Med order) | | | | | | | |
|  | **I.V. CONTRAST – 1^st^ injection** 40ml Iopamidol 370, unless alternate dose specified by Radiologist or due to patient Renal function (eGFR)  **2^nd^ injection** 90ml Iopamidol 370 | | | | | | | |
| **CT Technique**  **Without IV Contrast** | Kidneys to IT - 2.5mm Without - RENAL COLIC  LOW DOSE - AUTO mA - NOISE INDEX 40 | | | | | | | |
|  | kV | mA  **ASiR 40%** | Scan type  Rotation time | Scan  Thickness  (mm) | Scan mode | Speed  (mm) | Recon thickness  (mm) | Algo |
|  | 120 | 25-180 | Helical 0.5 | 2.5 | 0.984:1 | 39.37 | 2.5 | standard |
| **CT Technique**  **With IV Contrast** | Inject 40ml Iopamidol 370 2.5ml/sec and wait 5 min, rotate patient 180 to prone and back to supine  Scout at 8 min-if ureters opacified continue  If not, if not call Radiologist to assess to determine delay  Use Auto mA if possible for patient under 250 lbs.  NOISE INDEX 41.4  2^nd^ Scan-Inject 90cc Iopamidol 370 2.5ml/sec wait 100sec and scan Diaphragm to IT | | | | | | | |
|  | kV | mA  **ASiR 40%** | Scan type  Rotation time | Scan  Thickness  (mm) | Scan mode | Speed  (mm) | Recon thickness  (mm) | Algo |
|  | 120 | 100-450 | HELCIAL .8 | 1.25 | 0.984:1 | 39.37 | 2 AXIAL  2 CORONAL | STANDARD |
|  |  |  |  |  |  |  |  |  |
|  |  |  |  |  |  |  |  |  |
|  |  |  |  |  |  |  |  |  |
| **Comment** |  | | | | | | | |

# CT UROGRAPHY WITH INTRAVENOUS CONTRAST

| **Clinical Indication** | COMBINED PARENCHYMAL ORGAN AND PYELOUROGRAPHIC EVALUATION FOR NON STONE DISEASE SUCH AS F/U TRANSITIONAL CELL CA, ILEAL CONDUIT OR NEOBLADDER, BLADDER CA,URETERIC INJURY AND HEMATURIA | | | | | | | |
| --- | --- | --- | --- | --- | --- | --- | --- | --- |
| **Protocol Code** | **UROGRAM +** © | | | | | | | |
| **Contrast** | **ORAL CONTRAST –** H2O, 1X600ml 30 min prior to scan OR  If Patient unable to drink 250cc IV NS bolus before scan (requires Med order) | | | | | | | |
|  | **I.V. CONTRAST – 1^st^ injection** 40ml Iopamidol 370, unless alternate dose specified by Radiologist or due to patient Renal function (eGFR)  **2^nd^ injection** 90ml Iopamidol 370 | | | | | | | |
| **CT Technique**  **Without IV Contrast** |  | | | | | | | |
|  | kV | mA | Scan type  Rotation time | Scan  Thickness  (mm) | Scan mode | Speed  (mm) | Recon thickness  (mm) | Algo |
|  |  |  |  |  |  |  |  |  |
| **CT Technique**  **With IV Contrast** | Inject 40ml Iopamidol 370 2.5ml/sec and wait 8 min,  Scout, if ureters opacified continue  Use Auto mA if possible for patient under 250 lbs.  NOISE INDEX 41.4  Scan-Inject 90cc Iopamidol 370 2.5ml/sec wait 100sec and scan Diaphragm to IT | | | | | | | |
|  | kV | mA  **ASiR 40%** | Scan type  Rotation time | Scan  Thickness  (mm) | Scan mode | Speed  (mm) | Recon thickness  (mm) | Algo |
|  | 120 | 100-450 | HELCIAL .8 | 1.25 | 0.984:1 | 39.37 | 2 AXIAL  2 CORONAL | STANDARD |
|  |  |  |  |  |  |  |  |  |
|  |  |  |  |  |  |  |  |  |
|  |  |  |  |  |  |  |  |  |
| **Comment** |  | | | | | | | |

# ADRENAL LESION - AND RADIOLOGIST CHECK

| **Clinical Indication** | ADRENAL LESION | | | | | | | |
| --- | --- | --- | --- | --- | --- | --- | --- | --- |
| **Protocol Code** | **ADRENAL - AND CHECK if for PHEO include aortic bifurcation on PVP** | | | | | | | |
| **Contrast** | **ORAL CONTRAST –** No Drinks | | | | | | | |
|  | **I.V. CONTRAST –** **Will See** CHECK WITH RADIOLOGIST IF INDETERMINANT (i.e. if the adrenal nodule has ROI > 10HU you need to proceed with IV contrast) if unsure ask rad. | | | | | | | |
| **CT Technique**  **Without IV Contrast** | Adrenal Without - 2.5mm (approx - T12 level)  No auto mA- measuring adrenal same mA, KV slice thickness for all phases | | | | | | | |
|  | kV | mA | Scan type  Rotation time | Scan  Thickness  (mm) | Scan mode | Speed  (mm) | Recon thickness  (mm) | Algo |
|  | 120 | 300 | HELCIAL .8 | 2.5 | 0.984:1 | 39.37 | 2.5 AXIAL | STANDARD |
| **CT Technique**  **With IV Contrast** | PVP- Adrenals - 2.5mm -60 sec delay 2ml/sec  **if for PHEO include aortic bifurcation on PVP** | | | | | | | |
|  | kV | mA | Scan type  Rotation time | Scan  Thickness  (mm) | Scan mode | Speed  (mm) | Recon thickness  (mm) | Algo |
|  | 120 | 300 | HELCIAL .8 | 2.5 | 0.984:1 | 39.37 | 2.5 AXIAL | STANDARD |
|  | Delayed adrenals - 2.5mm -10 min delay | | | | | | | |
|  | kV | mA | Scan type  Rotation time | Scan  Thickness  (mm) | Scan mode | Speed  (mm) | Recon thickness  (mm) | Algo |
|  | 120 | 300 | HELCIAL .8 | 2.5 | 0.984:1 | 39.37 | 2.5 AXIAL | STANDARD |
| **Comment** | Call rad after w/o to see if contrast is required.  Doctor may add a complete abd 5mm to the PVP in addition to adrenals | | | | | | | |

CT CYSTOGRAM

| **Clinical Indication** | | Urethral or bladder rupture, pelvic trauma, small bladder lesions (could be used as virtual cystoscopy) | | | | | | | |
| --- | --- | --- | --- | --- | --- | --- | --- | --- | --- |
| **Protocol Code** | | **CT CYSTOGRAM** | | | | | | | |
| **Contrast** | | NO Oral or IV contrast is required  Foley’s catheter | | | | | | | |
|  |  | Contrast preparation: 30cc of Iopamidol 370 in a 500cc bag of normal saline.  The bag is connected to the Foley’s Catheter and drips under gravity | | | | | | | |
| **CT Technique**  **Without IV Contrast** | | Please do not start scan until ~ 200-300cc of mixture as been infused.  Use Auto mA if possible  NOISE INDEX 41.4 | | | | | | | |
|  | | kV | mA | Scan type  Rotation time | Scan  Thickness  (mm) | Scan mode | Speed  (mm) | Recon thickness  (mm) | Algo |
|  |  | 120 | 100-400 | HELCIAL .8 | 1.25 | 0.984:1 | 39.37 | 2.5 AXIAL  3 COR | STANDARD |
| **CT Technique**  **With IV Contrast** | |  | | | | | | | |
|  |  | kV | mA | Scan type  Rotation time | Scan  Thickness  (mm) | Scan mode | Speed  (mm) | Recon thickness  (mm) | Algo |
|  |  |  |  |  |  |  |  |  |  |
|  |  |  |  |  |  |  |  |  |  |
|  | | kV | mA | Scan type  Rotation time | Scan  Thickness  (mm) | Scan mode | Speed  (mm) | Recon thickness  (mm) | Algo |
|  | |  |  |  |  |  |  |  |  |
|  | |  | | | | | | | |
| **Comment** | |  | | | | | | | |

# APPENDIX WITH INTRAVENOUS CONTRAST

| **Clinical Indication** | High suspicion of appendicitis with or without indeterminate US | | | | | | | |
| --- | --- | --- | --- | --- | --- | --- | --- | --- |
| **Protocol Code** | **APPENDIX**+© | | | | | | | |
| **Contrast** | ORAL CONTRAST – Not required | | | | | | | |
|  | I.V. CONTRAST – 80 –100ml Iopamidol 370, unless alternate dose specified by Radiologist. | | | | | | | |
| **CT Technique**  **Without IV Contrast** |  | | | | | | | |
|  | kV | mA | Scan type  Rotation time | Scan  Thickness  (mm) | Scan mode | Speed  (mm) | Recon  Thickness  (mm) | Algo |
|  |  |  |  |  |  |  |  |  |
| **CT Technique**  **With IV Contrast** | PVP- L2 to Symphysis Pubis– 5mm - 80 sec delay 2 ml/sec  Use dose modulation if possible for patient under 250 lbs.  Noise Index 47 | | | | | | | |
|  | kV | mA | Scan type  Rotation time | Scan  Thickness  (mm) | Scan mode | Speed  (mm) | Recon  Thickness  (mm) | Algo |
|  | 120 | 100-400 | HELICAL .5 | 0.625 | 0.984:1 | 39.37 | 2 AXIAL  3 CORONAL | STANDARD |
|  |  |  |  |  |  |  |  |  |
|  | kV | mA | Scan type  Rotation time | Scan  Thickness  (mm) | Scan mode | Speed  (mm) | Recon thickness  (mm) | Algo |
|  |  |  |  |  |  |  |  |  |
| **Comment** | The default for patients < 35 years, and < 130 lbs would be 100KV. | | | | | | | |

SMALL BOWEL OBSTRUCTION WITH INTRAVENOUS CONTRAST

| **Clinical Indication** | Small bowel obstruction (SBO) suspected or in known SBO in patients to help define surgical versus none surgical management | | | | | | | |
| --- | --- | --- | --- | --- | --- | --- | --- | --- |
| **Protocol Code** | **SBO + ©** | | | | | | | |
| **Contrast** | **ORAL CONTRAST – 9%** Diatrizoate Meglumine short, 1X600ml  Must wait at least 3 hours before scanning | | | | | | | |
|  | **I.V. CONTRAST –** 80 –100ml Iopamidol 370, unless alternate dose specified by Radiologist. | | | | | | | |
| **CT Technique**  **Without IV Contrast** |  | | | | | | | |
|  | kV | mA | Scan type  Rotation time | Scan  Thickness  (mm) | Scan mode | Speed  (mm) | Recon thickness  (mm) | Algo |
|  |  |  |  |  |  |  |  |  |
| **CT Technique**  **With IV Contrast** | PVP-Abdomen and pelvis – 5mm - 65 sec delay  Use Auto mA if possible for patient under 250 lbs.  NOISE INDEX 41.4 | | | | | | | |
|  | kV | mA  **ASiR 40%** | Scan type  Rotation time | Scan  Thickness  (mm) | Scan mode | Speed  (mm) | Recon thickness  (mm) | Algo |
|  | 120 | 100-450 | HELCIAL .8 | 1.25 | 0.984:1 | 39.37 | 2.5 AXIAL  2 CORONAL | STANDARD |
|  |  |  |  |  |  |  |  |  |
|  | kV | mA | Scan type  Rotation time | Scan  Thickness  (mm) | Scan mode | Speed  (mm) | Recon thickness  (mm) | Algo |
|  |  |  |  |  |  |  |  |  |
| **Comment** |  | | | | | | | |

HERNIA EVALUATION

| **Clinical Indication** | Ultrasound FIRST LINE EXCEPT WHEN LARGE BMI OR SURGICAL PLANNING | | | | | | | |
| --- | --- | --- | --- | --- | --- | --- | --- | --- |
| **Protocol Code** | **HERNIA** | | | | | | | |
| **Contrast** | **ORAL CONTRAST** – 2% Telebrix Long, 2X600ml, 1/2cup at O min, 30 min, 60 min, 90 min, Make sure to have some on the table to fill stomach | | | | | | | |
|  | **I.V. CONTRAST –** No I.V. Contrast  ***If there is concern for strangulation, IV contrast phase can be added.  80 –100ml Iopamidol 370, 2 ml/sec unless alternate dose specified by Radiologist. | | | | | | | |
| **CT Technique**  **Without IV Contrast** | Abdomen and Pelvis WITHOUT – 5mm  WITH VALSAVA TECHNIQUE  Use Auto mA if possible for patient under 250 lbs.  NOISE INDEX 41.4 | | | | | | | |
|  | kV | mA  **ASiR 40%** | Scan type  Rotation time | Scan  Thickness  (mm) | Scan mode | Speed  (mm) | Recon thickness  (mm) | Algo |
|  | 120 | 200-450 | HELICAL .8 | 1.25 | 0.984:1 | 39.37 | 5 AXIAL  2 CORONAL | STANDARD |
| **CT Technique**  **With IV Contrast** | ? STRANGULATION – THEN ADD Abdomen and Pelvis + c | | | | | | | |
|  | kV | mA | Scan type  Rotation time | Scan  Thickness  (mm) | Scan mode | Speed  (mm) | Recon thickness  (mm) | Algo |
|  |  |  |  |  |  |  |  |  |
|  |  |  |  |  |  |  |  |  |
|  | kV | mA | Scan type  Rotation time | Scan  Thickness  (mm) | Scan mode | Speed  (mm) | Recon thickness  (mm) | Algo |
|  |  |  |  |  |  |  |  |  |
| **Comment** |  | | | | | | | |

**MESENTERIC ANGIOGRAM WITH AND WITHOUT INTRAVNEOUS CONTRAST**

| **Clinical Indication** | BOWEL ISCHEMIA, ACUTE GI BLEED OR HIGH RISK ANEMIA | | | | | | | |
| --- | --- | --- | --- | --- | --- | --- | --- | --- |
| **Protocol Code** | **CTA MESENTERIC +/-©** | | | | | | | |
| **Contrast** | **ORAL CONTRAST –** NO ORAL CONTRAST unless high risk anemia then give 1.5 L PEG | | | | | | | |
|  | I.V. CONTRAST – 80 –110ml Iopamidol 370, unless alternate dose specified by Radiologist. | | | | | | | |
| **CT Technique**  **Without IV Contrast** | Abdomen/Pelvis Without – 5mm  Use Auto mA if possible for patient under 250 lbs.  NOISE INDEX 27 | | | | | | | |
|  | kV | mA  **ASiR 40%** | Scan type  Rotation time | Scan  Thickness  (mm) | Scan mode | Speed  (mm) | Recon thickness  (mm) | Algo |
|  | 120 | 80-400 | HELICAL .8 | 5 | 0.984:1 | 39.37 | 5 AXIAL | STANDARD |
| **CT Technique**  **With IV Contrast** | ART Phase - Abdomen and Pelvis - 2.5mm  BOLUS TRACKING - 15 sec from 100 HU 2.5ml/sec  NOISE INDEX 41.4 | | | | | | | |
|  | kV | mA  **ASiR 40%** | Scan type  Rotation time | Scan  Thickness  (mm) | Scan mode | Speed  (mm) | Recon thickness  (mm) | Algo |
|  | 120 | 80-600 | HELICAL .5 | 0.625 | 0.984:1 | 39.37 | 2.5 AXIAL  5 COR MIP  5 SAG MIP | STANDARD |
|  | PVP –Abdomen and Pelvis – 5mm  BOLUS TRACKING- 65 sec from 100 HU  NOISE INDEX 41.4 | | | | | | | |
|  | kV | mA  **ASiR 40%** | Scan type  Rotation time | Scan  Thickness  (mm) | Scan mode | Speed  (mm) | Recon thickness  (mm) | Algo |
|  | 120 | 100-450 | HELICAL .5 | 1.25 | 0.984:1 | 39.37 | 5 AXIAL  3 COR | STANDARD |
| **Comment** | Coronal and Sag MIP- Art Phase  Coronal MPR 3mm –PV Phase | | | | | | | |

**PERITONEAL DIALYSIS CATHETER LEAK**

| **Clinical Indication** | SUSPECTED PERITONEAL DIALYSIS LEAK | | | | | | | |
| --- | --- | --- | --- | --- | --- | --- | --- | --- |
| **Protocol Code** | **PD CATHETER LEAK** | | | | | | | |
| **Contrast** | **ORAL CONTRAST –** No Drinks | | | | | | | |
|  | **I.V. CONTRAST –** No I.V. Contrast | | | | | | | |
|  | CATHETER CONTRAST – 50 ml Iopamidol 370, unless alternate dose specified by Radiologist. Use Luer Lock Syringe Pt to be NPO for duration of exam | | | | | | | |
| **CT Technique**  **Without IV Contrast** | 1. Dialysis nurse should drain abdomen 2. PD Nurse injects 50 cc Iopamidol 370 into PD Fluid and infuses into abdomen 3. Scan patient immediately Abdomen and Pelvis 4. Scan Abdomen and Pelvis 2 hours later- roll patient several times just before scanning   Use Auto Ma if possible for patients under 250 lbs  Noise index 16-20 | | | | | | | |
|  | kV | mA | Scan type  Rotation time | Scan  Thickness  (mm) | Scan mode | Speed  (mm) | Recon thickness  (mm) | Algo |
|  | 120 | 300 | Helical .8 | 5 | 1.5 :1 | 15 | 5 | standard |
| **CT Technique**  **With IV Contrast** |  | | | | | | | |
|  | kV | mA | Scan type  Rotation time | Scan  Thickness  (mm) | Scan mode | Speed  (mm) | Recon thickness  (mm) | Algo |
|  |  |  |  |  |  |  |  |  |
|  |  |  |  |  |  |  |  |  |
|  |  |  |  |  |  |  |  |  |
|  |  |  |  |  |  |  |  |  |
| Comment | PD Dialysis involved  Technologist to include scrotum on all scans  Show images to Radiologist before patient goes back to Dialysis to be drained | | | | | | | |

# CT ENEROGRAPHY WITH INTRAVENOUS CONTRAST

| **Clinical Indication** | CROHN’S ACTIVITY | | | | | | | |
| --- | --- | --- | --- | --- | --- | --- | --- | --- |
| **Protocol Code** | **CTE +© PEG (or other oral contrast to be specified)** | | | | | | | |
| **Contrast** | **ORAL CONTRAST –** POLYETHYLENE GLYCOL 1 – 1.5 litres 500 cc q 20min x 2, and H20 450cc on table. Pt should continue with third cup of PEG if there is a delay in getting scan done.  Or as specified by radiologist | | | | | | | |
|  | **I.V. CONTRAST –** 90 –110ml Iopamidol 370, unless alternate dose specified by Radiologist. | | | | | | | |
| **CT Technique**  **Without IV Contrast** |  | | | | | | | |
|  | kV | mA | Scan type  Rotation time | Scan  Thickness  (mm) | Scan mode | Speed  (mm) | Recon thickness  (mm) | Algo |
|  |  |  |  |  |  |  |  |  |
| **CT Technique**  **With IV Contrast** | PORTAL VENOUS PHASE- Abdomen and Pelvis – 2.5mm  3.2 ml/sec– Iopamidol 370  Use Auto mA if possible for patient under 250 lbs.  45sec enteric phase  NOISE INDEX 41.4 | | | | | | | |
|  | kV | mA  **ASiR 40%** | Scan type  Rotation time | Scan  Thickness  (mm) | Scan mode | Speed  (mm) | Recon thickness  (mm) | Algo |
|  | 120 | 100-450 | HELCIAL .8 | 0.625 | 0.984:1 | 39.37 | 2.5 AXIAL  2 CORONAL | STANDARD |
|  |  |  |  |  |  |  |  |  |
|  | kV | mA | Scan type  Rotation time | Scan  Thickness  (mm) | Scan mode | Speed  (mm) | Recon thickness  (mm) | Algo |
|  |  |  |  |  |  |  |  |  |
| **Comment** | The default for patients < 150 lbs would be 100KV. | | | | | | | |

# CT ENTEROGRAPHY FORSTRICTURE EVALUATION IN EMERGENCY DEPARTMENT

| **Clinical Indication** | STRICTURE EVALUATION IN PATIENT WITH KNOWN CROHN’S DISEASE OR OTHER CAUSES OF RECURRENCE SBO | | | | | | | |
| --- | --- | --- | --- | --- | --- | --- | --- | --- |
| **Protocol Code** | **CTE +© 9%** | | | | | | | |
| **Contrast** | **ORAL CONTRAST –** 9% DIATRIZOATE MEGLUMINE – 1 litre; 250 cc q 15min x 4  Or as specified by radiologist | | | | | | | |
|  | **I.V. CONTRAST –** 90 –110ml Iopamidol 370, unless alternate dose specified by Radiologist. | | | | | | | |
| **CT Technique**  **Without IV Contrast** | Abdomen and Pelvis **PRONE**– 2.5mm  **Verify oral contrast has reached Terminal Ileum before supine.**  NOISE INDEX 64 | | | | | | | |
|  | kV | mA  **ASiR 40%** | Scan type  Rotation time | Scan  Thickness  (mm) | Scan mode | Speed  (mm) | Recon thickness  (mm) | Algo |
|  | 120 | 100-300 | HELCIAL .8 | 0.625 | 0.984:1 | 39.37 | 2.5 AXIAL  2 CORONAL | STANDARD |
| **CT Technique**  **With IV Contrast** | PVP- Abdomen and Pelvis **SUPINE** – 2.5mm  3.2 ml/sec– Iopamidol 370  65sec delay  NOISE INDEX 41.4 | | | | | | | |
|  | kV | mA  **ASiR 40%** | Scan type  Rotation time | Scan  Thickness  (mm) | Scan mode | Speed  (mm) | Recon thickness  (mm) | Algo |
|  | 120 | 100-450 | HELCIAL .8 | 0.625 | 0.984:1 | 39.37 | 2.5 AXIAL  2 CORONAL  15 CORMIP | STANDARD |
|  |  |  |  |  |  |  |  |  |
|  | kV | mA | Scan type  Rotation time | Scan  Thickness  (mm) | Scan mode | Speed  (mm) | Recon thickness  (mm) | Algo |
|  |  |  |  |  |  |  |  |  |
| **Comment** | The default for patients < 150 lbs would be 100KV.  CORONAL 15mm MIP OF THE PVP PHASE | | | | | | | |

# CT ENTEROGRAPHY FOR ANEMIA EVALUATION WITH AND WITHOUT INTRAVNEOUS CONTRAST

| **Clinical Indication** | ANEMIA | | | | | | | |
| --- | --- | --- | --- | --- | --- | --- | --- | --- |
| **Protocol Code** | **CTE +/v-© PEG DUAL ENERGY** | | | | | | | |
| **Contrast** | **ORAL CONTRAST –** PEG 1 – 1.5 litres 500 cc q 20min x 2, and H20 450cc on table. Pt should continue with third cup of PEG if there is a delay in getting scan done.  Or as specified by radiologist | | | | | | | |
|  | **I.V. CONTRAST –** 90 –110ml Iopamidol 370, unless alternate dose specified by Radiologist. | | | | | | | |
| **CT Technique**  **Without IV Contrast** |  | | | | | | | |
|  | kV | mA | Scan type  Rotation time | Scan  Thickness  (mm) | Scan mode | Speed  (mm) | Recon thickness  (mm) | Algo |
|  |  |  |  |  |  |  |  |  |
| **CT Technique**  **With IV Contrast** | Abdomen and Pelvis – 2.5mm  3.2 ml/sec– Iopamidol 370  45sec enteric phase  70 sec portal venous phase (DE with full GSI dataset) | | | | | | | |
|  | kV | mA  **ASiR 40%** | Scan type  Rotation time | Scan  Thickness  (mm) | Scan mode | Speed  (mm) | Recon thickness  (mm) | Algo |
|  | 100 |  | HELCIAL | 0.625 | 0.984:1 | 39.37 | 2.5 AXIAL  2 CORONAL | STANDARD |
|  |  |  |  |  |  |  |  |  |
|  | kV | mA  **ASiR 40%** | Scan type  Rotation time | Scan  Thickness  (mm) | Scan mode | Speed  (mm) | Recon thickness  (mm) | Algo |
|  | GSI |  | HELCIAL .8 | 0.625 | 0.984:1 | 39.37 | 2.5 AXIAL  2 CORONAL | STANDARD |
| **Comment** | GSI PVP: 75KeV, 140KeV, MD Water(Iodine)+Iodine(Water), MSI (Iodine subtraction) | | | | | | | |

>48 HOURS POST-OP BARIATRIC SURGERY WITH INTRAVENOUS CONTRAST

| **Clinical Indication** | Weeks or months post operative, looking for late complications, such as pouch obstruction, fistula, SBO and internal hernia | | | | | | | |
| --- | --- | --- | --- | --- | --- | --- | --- | --- |
| **Protocol Code** | **>48h POST-OP** **BARIATRIC -Short Oral** | | | | | | | |
| **Contrast** | **ORAL CONTRAST –** 9% Diatrizoate Meglumine short, 250 ml maximum. Plus oral on the table. | | | | | | | |
|  | I.V. CONTRAST – 80-100 ml Iopamidol 370 or as specified by Radiologist | | | | | | | |
| **CT Technique**  **Without IV Contrast** |  | | | | | | | |
|  | kV | mA | Scan type  Rotation time | Scan  Thickness  (mm) | Scan mode | Speed  (mm) | Recon  Thickness  (mm) | Algo |
|  |  |  |  |  |  |  |  |  |
| **CT Technique**  **With IV Contrast** | Below tracheal bifurcation to IT (Abdomen and Pelvis)  **- oral contrast**  NOISE INDEX 36.5 Regular dose | | | | | | | |
|  | kV | mA | Scan type  Rotation time | Scan  Thickness  (mm) | Scan mode | Speed  (mm) | Recon thickness  (mm) | Algo |
|  | 120 | 100-450  NI 36.5 | HELICAL 0.8 | 1.25 | 0.984:1 | 39.37 | 5 AXIAL  3 COR | STANDARD |
|  |  |  |  |  |  |  |  |  |
|  | kV | mA | Scan type  Rotation time | Thickness  (mm) | Scan mode | Speed  (mm) | Recon thickness  (mm) | Algo |
|  |  |  |  |  |  |  |  |  |
| **Comment** | To be used when pt presents to ER weeks or months post operative, this is usually looking for late complications like internal hernias. | | | | | | | |

# SUSPECTED HYPERVASC METASTASES WITH AND WITHOUT INTRAVENOUS CONTRAST

| **Clinical Indication** | Staging or f/u specifically for patients with melanoma, GIST, RCC, NET or other hypervascular metastases. | | | | | | | |
| --- | --- | --- | --- | --- | --- | --- | --- | --- |
| **Protocol Code** | HYPERVASC METS +/- © | | | | | | | |
| **Contrast** | **ORAL CONTRAST –** No Oral contrast unless specified. Can ADD LONG drinks on request by radiologist especially for Gastrointestinal Stromal Tumor. Can dd H20 Oral on request for any of these tumors. | | | | | | | |
|  | **I.V. CONTRAST –** 90 –110ml Iopamidol 370, unless alternate dose specified by Radiologist. | | | | | | | |
| **CT Technique**  **Without IV Contrast** | Abdomen – 5mm – Without  Use Auto mA if possible for patient under 250 lbs.  NOISE INDEX 30 | | | | | | | |
|  | kV | mA  **ASiR 40%** | Scan type  Rotation time | Scan  Thickness  (mm) | Scan mode | Speed  (mm) | Recon thickness  (mm) | Algo |
|  | 120 | 100-400 | HELCIAL .8 | 5 | 0.984:1 | 39.37 | 5 | STANDARD |
| **CT Technique**  **With IV Contrast** | Abdomen and Pelvis - 2.5mm  Arterial phase - BOLUS TRACKING- 20 sec (HAP) from 100 HU  3.2ml/sec  NOISE INDEX 41.4 | | | | | | | |
|  | kV | mA  **ASiR 40%** | Scan type  Rotation time | Scan  Thickness  (mm) | Scan mode | Speed  (mm) | Recon thickness  (mm) | Algo |
|  | 120 | 100-500 | HELCIAL .8 | 0.625 | 0.984:1 | 39.37 | 2 AXIAL  2 CORONAL  2 SAG | STANDARD |
|  | PVP – Abdomen and Pelvis – 5mm - 50 sec from 100 HU | | | | | | | |
|  | kV | mA  **ASiR 40%** | Scan type  Rotation time | Scan  Thickness  (mm) | Scan mode | Speed  (mm) | Recon thickness  (mm) | Algo |
|  | 120 | 100-400 | HELCIAL .8 | 1.25 | 0.984:1 | 39.37 | 5 AXIAL | STANDARD |
| **Comment** |  | | | | | | | |
